# Correcting loss of a papilla following orthodontic space opening (Atherton´s patch) through implant supported rehabilitation. A case report

**DOI:** 10.4317/jced.51281

**Published:** 2014-02-01

**Authors:** Jose Viña, Jose Balaguer, Luis Martorell, Miguel Peñarrocha

**Affiliations:** 1 Master of Oral Surgery and Implantology, Valencia University Medical and Dental School, Valencia, Spain; 2 Associate Professor of Oral Surgery. Professor of the Master in Oral Surgery and Implantology. Faculty of Medicine and Dentistry. University of Valencia. Valencia. Spain; 3 Chairman of Oral Surgery, Director of the Master of Oral Surgery and Implantology, Valencia University Medical and Dental School, Valencia, Spain

## Abstract

The objective of this case report is to describe a surgical and prosthetic technique to create a lost papilla following orthodontic space opening (Atherton´s patch) through implant supported rehabilitation. A switching platform implant was used to replace a left maxillary canine in a unitary interdental edentulous ridge with Atherton´s patch in the distal area of the upper lateral left incisor. The radiographic study revealed correct level of the interproximal bone of the adjacent teeth. A mucoperiosteal flap with crest incision and sulcular extension to the adjacent teeth was made. Special attention was paid to correct position of the implant and the distance (≥ 1.5 mm) between the platform and the roots of the adjacent teeth. A submerged technique was used. Tissue modeling through provisional crown was performed in order to create an ideal emergence profile with total papilla fill recorded at the Atherton´s patch area. Final screw retained CAD-CAM zirconia structure was place. Final follow up was performed 2 years after provisional crown placement, and total fill of both papilla, including at Atherton´s patch area, was recorded.

** Key words:**Atherton´s patch, papilla, switching platform, implant and orthodontics, esthetic score.

## Introduction

Atherton’s patch is the stretch of the gingival sulcus, creating a gingival depression, in the tension area to the orthodontically moved tooth (1). During orthodontic site development, the interproximal papilla remains adjacent to the tooth that is not moving (1). This interproximal papilla is a parameter of great importance from esthetic point of view in implant supported rehabilitations (2).

Kokich (3) proposed an advance flap to create the papilla adjacent to an implant in an edentulous space with Atherton´s patch. The technique consisted on placing a 2 mm healing abutment following implant placement, and using a submerged technique. Choquet et al. (4) performed a study where found that, between an implant and a tooth, a papilla will form in 100% of the cases, if there were 5 mm or less between the interproximal pick of the bone and the contact point of the implant restoration and the tooth.

To our knowledge, no clinical studies or case report has been performed in order to describe implant rehabilita-tions at Atherton´s patch areas. So, the objective of this case report is to describe a surgical and prosthetic technique to create a lost papilla following orthodontic space opening (Atherton´s patch) through implant supported rehabilitation.

## Case Report

- Treatment plan

**Figure 1:**
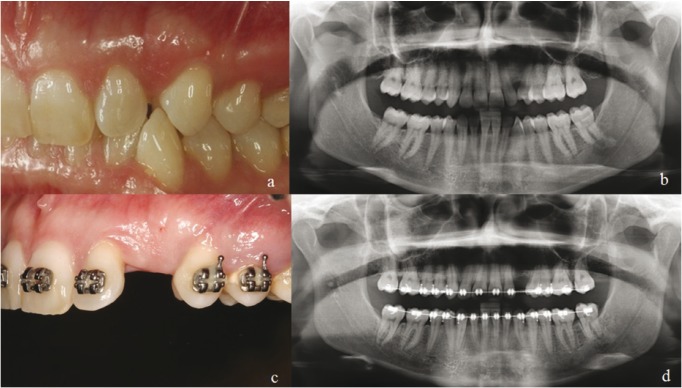
Clinical and radiografic examination. a) Intraoral view before orthodontic treatment with the absence of the left upper canine. b) Radiografic examination. Note the correct level of the interproximal bone of the adjacent teeth. c) Intraoral view after after interdental espace opening. Enough mesio-distal space was present. The Atherton´s patch is present at the distal aspect of the left upper lateral incisor. d) The panoramic radiographs shows correct level of the interproximal bone of the adjacent teeth.

A 35 year-old female nonsmoker, without systemic diseases that could alter the tissue integration of dental implants presented for treatment in a university dental clinic. The patient had absence of the left upper canine (impacted tooth extracted in the past), and treatment plan involved orthodontic site development and implant supported rehabilitation (Fig. 1). After orthodontic treatment, where the left upper lateral incisor was moved mesially to open ideal space for implant restoration, clinical examination showed an Atherton´s patch at the distal area of the lateral incisor (Fig. 1). Enough mesio-distal space and bucco-palatal width were recorded. Radiographic examinations showed no interproximal bone loss adjacent to the teeth (Fig. 1).

- Surgical procedure

**Figure 2:**
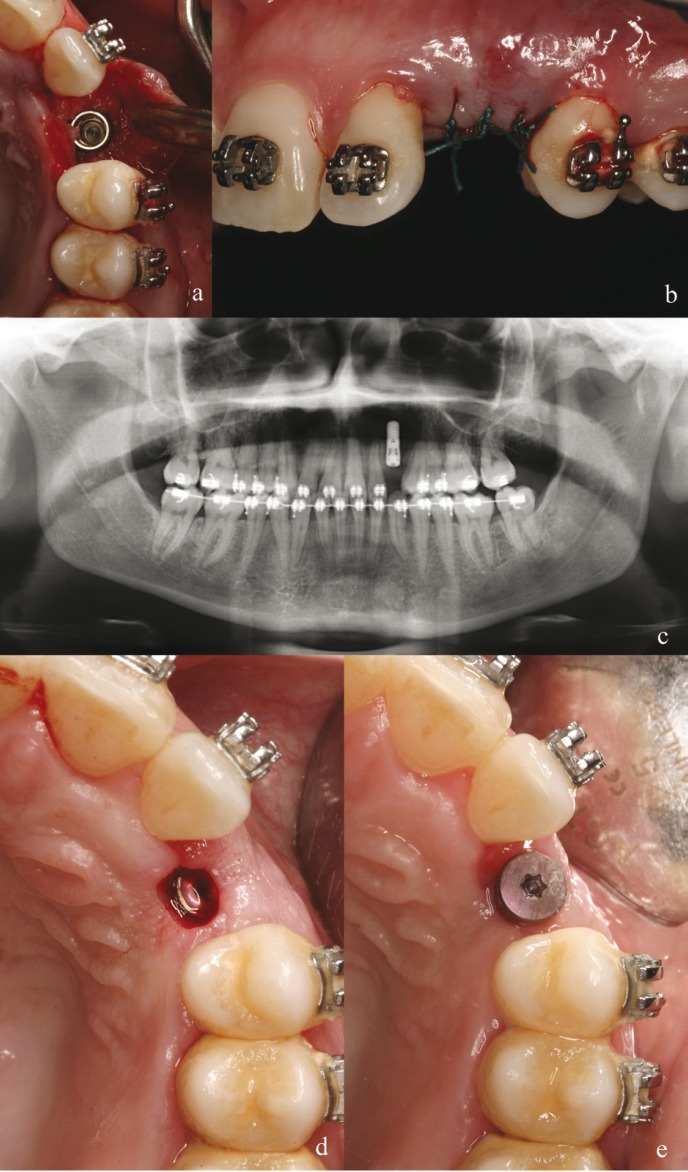
Implant surgery. a) Mucoperiosteal flap with mid crest incision and intrasulcular extensions and ideal three dimension implant placement. b) Submerged technique was carried out. c) Panoramic radiographs after implant placement. d) View of the second stage surgery with circular incision slighly palatal to the implant platform. e) Soft tissue after 2 weeks of healing period.

Implant surgery was carried out with local anesthesia, (4% articaine and adrenalin 1:100,000; Ultracain®, Aventis Pharma, Bad Soden, Germany). The flap design consisted of mucoperiosteal flap with mid crest incision and intrasulcular extensions of the adjacent teeth (Fig. 2). The implant used was a bone-level implant featuring a chemically modified, sandblasted and acid-etched surface in the endosseous portion, with a platform diameter of 4.1 mm and a length of 12 mm. (Straumann Bone Level SLActive® Basel, Switzerland.) An ideal three dimension implant placement was carefully carried out. Special attention was paid to place the implant platform around 2 mm far from interproximal aspect from adjacent teeth (the presence of this bone is going to support the future papilla). To minimize peri-implant bone remodeling, switching platform concept implant was used. Submerged technique was performed with nonabsorbable sutures (Fig. 2). Perioperative antibiotic prophylaxis was initiated 12 hours prior to surgery and maintained one week postsurgically (amoxicillin 500 mg. 3 times a day for 7 days). Post-surgical medication also included Ibuprofen 600 mg. 3 times a day for 2 days, and chlorhexidine digluconate (0.1%) rinse 2 times per day for 10 days. Sutures were removed after 1 week. Second surgery was performed 3 month later with circular incision slightly palatal to the implant platform with the aim to push the soft tissue buccally (Fig. 2).

- Restorative procedure

**Figure 3:**
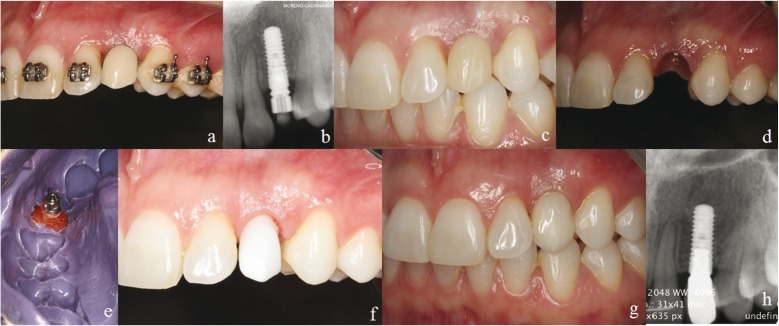
Restorative procedure. a) Screw-retained provisional acrylic crowns was used to performe the peri-implant soft tissue conditioning phase. Note the total absecnce of mesial papilla. Around 2 to 3 mm of root cementum of the distal aspect of the left upper lateral incisor is clinically visible. b) Periapical radiographs of the implant loaded. Note the correct level of the interproximal bone at the adjacent teeth. c) After 6 month, complete papilla fill was recorded. d) Emegence profile the day of the final impressions. Note the scalloped image. e) Customized impression of the implant and the sof tissue. f) Zirconia structure design CAD/CAM technology. g) Final direct screw rehabilitation was inserted. Note the papilla fill. h) Periapical radiographs with the final restoration. Interproximal bone level are manteined.

Impressions were taken, 2 weeks later, using close tray technique, and screw-retained provisional acrylic crowns were inserted to initiate the peri-implant soft tissue conditioning phase. Special care was conducted on the pres-sure on the peri-implant mucosa, and contact points between implant restoration and adjacent teeth. At this moment, total absence of papilla was recorded, being this absence more pronounced at the Atherton´s patch area. At this moment, around 2 to 3 mm of root cementum of the distal aspect of the left upper lateral incisor was clinically visible (Fig. 3). Periapical radiograph showed correct level of the interproximal bone at the adjacent teeth (Fig. 3). Two and four month later, the provisional crown was enlarged, to optimize the papilla fill. Six month after abutment connection, complete papilla formation was recorded both the distal and at the Atherton´s patch area (Fig. 3), and a scalloped profile was present (Fig. 3).

Final impressions were taken using open tray technique. Customized impression post (using Duralay resin) was used to record the emergence profile (Fig. 3). Direct screw retained zirconia structure was design and produce using CAD/CAM technology (Fig. 3). Final direct screw rehabilitation was inserted and papilla fill was recorded at this point (Fig. 3).

- Follow up

Radiographic study did not reveal any signs of continuous peri-implant radiolucency throughout the observation period (3,6,12 month). Final follow up was carried out 18 month after definitive restoration placement. Stability of hard and soft tissues was recorded.

## Discussion

The aim of the present case report was to describe a surgical and prosthetic procedure to achieve papilla forma-tion in an edentulous single implant restoration with Atherton´s patch. At the end of the treatment, papilla formation was reach. In this case, the treatment was performed in an adult woman. Kokich (3) pointed that the age of the patient is an important factor in relation with Atherton´s patch management. If the patient remains growth potential (young), papilla formation after orthodontic treatment is predictable, but in adults patients without tooth eruption potential, papilla will not be formed.

In order to achieve papilla formation in adult patients with Atherton´s patch, Kokich (3) proposed a surgical technique including an advance flap to cover the implant. The technique consisted on placing a 2 mm healing abutment following implant placement, and using a submerged technique. With this surgical approach, he reached papilla formation. In the present case report, a tension free flap was used to submerge the implant, and in the provisional restorative phase attention was paid in the distance between the contact point and the pick of bone next to the adjacent tooth.

The level of the papilla is independent of the proximal bone level next to the implant, but is related to the inter-proximal bone level next to the adjacent tooth (5,6). Thus, the peak of interproximal bone determines the level of papilla. A distance of 1.5 mm between tooth and implant is necessary to maintain the interproximal height of the bone after remodeling of the biologic width (7). If there are 5 mm or less between the interproximal pick of the bone and the contact point of the implant restoration and the tooth, a papilla will form in 100% of the cases (4). In the present case enough mesio-distal space and correct three dimensional positioning of the implant (8) was achieved, so maintenance of the distal pick of bone of the lateral incisor could be achieved. Moreover using switching platform implant, the peri-implant bone remodeling diminishes compare with straight platform implants. This factor is important in order to preserve the interproximal pick of bone of adjacent teeth (9). Lee et al. (10) also observed between adjacent implants, that the dimension of the keratinized mucosa (mucogingival junction-pick of papilla) was related with the dimension of the papilla.

Different surgical techniques to generate interproximal papilla have been tried (11-14), but because no long-term studies have been conducted, no particular technique is recommended over another (15).

A clinical case report where papilla formation was achieved at an Atherton´s patch area was presented. The surgical and restorative treatment are explained and discussed. The present clinical case shows that if interproximal pick of bone at the adjacent teeth is present, enough mesio-distal space exists, and correct implant positioning is achieved, papilla will form in cases with Atherton´s patch.
